# Quantification of Aortic Valve Calcifications Detected During Lung Cancer-Screening CT Helps Stratify Subjects Necessitating Echocardiography for Aortic Stenosis Diagnosis

**DOI:** 10.1097/MD.0000000000003710

**Published:** 2016-05-13

**Authors:** Hee Young Lee, Sung Mok Kim, Kyung Soo Lee, Seung Woo Park, Myung Jin Chung, Hyoun Cho, Jung Im Jung, Hye Won Jang, Sin-Ho Jung, Juna Goo

**Affiliations:** From the Department of Radiology (HYL, SMK, KSL, MJC) and Division of Cardiology (SWP), Department of Medicine, Samsung Medical Center, Sungkyunkwan University School of Medicine; Department of Radiology, Seoul Metropolitan Government-Seoul National University Boramae Medical Center (HC); Department of Radiology (JIJ), Seoul St Mary's Hospital, College of Medicine, The Catholic University of Korea; Department of Medical Education (HWJ), Samsung Medical Center, Sungkyunkwan University School of Medicine; Biostatistics and Clinical Epidemiology Center (SHJ, JG), Samsung Medical Center, Sungkyunkwan University School of Medicine, Seoul, Korea.

## Abstract

No study has been published on aortic valve calcification (AVC) extent at lung cancer screening low-dose CT (LDCT) and its relationship with aortic stenosis (AS). The purpose of this study was to estimate the cutoff value of AVC on LDCT for detecting AS in asymptomatic Asian subjects. Six thousand three hundred thirty-eight subjects (mean age, 55.9 years ± 8.6) self-referred to health-promotion center underwent LDCT, coronary calcium scoring CT (CSCT), and echocardiography. AVC was quantified using Agatston methods on CT. AVC extent on LDCT was compared with that on CSCT, and AVC threshold for diagnosing AS was calculated. Clinical factors associated with AS and AVC were sought.

AVC was observed in 403 subjects (64.9 years ± 8.7) on LDCT (6.4%), and AVC score measured from LDCT showed strong positive correlation with that from CSCT (*r* = 0.83, *P* < 0.0001). Of 403 subjects, 40 (10%) were identified to have AS on echocardiography. Cutoff value of AVC score for detecting AS was 138.37 with sensitivity of 90.0% and specificity 83.2%. On multivariate analysis, age (odds ratio [OR] = 1.10, 95% CI: 1.09–1.12) and hypertension (OR = 1.39, 95% CI: 1.10–1.76) were associated with the presence of AVC, whereas AVC extent at LDCT (OR = 104.32, 95% CI: 16.16–673.70) was the only significant clinical factor associated with AS; AVC extent on LDCT (OR = 104.32, 95% CI: 16.16–673.70) was the significant clinical factor associated with AS.

The AVC extent on LDCT is significantly related to the presence of AS, and we recommend echocardiography for screening AS based on quantified AVC values on LDCT.

## Abstract 2

Supplemental Digital Content is available in the text

## INTRODUCTION

Smoking is a major risk factor for both cardiovascular disease and lung cancer, and cardiovascular disease is a major cause of morbidity and mortality worldwide, particularly in heavy smokers.^1,2^ Lung cancer screening CT using low-dose CT (LDCT), which is recommended in high-risk heavy smokers (>30 pack years and <15 years after smoking cessation), proved to be effective in reducing cancer-related mortality.^3^ Because CT scanners have advanced in terms of gantry rotation speed and spatial and temporal resolution, both pulmonary and cardiac diseases can be evaluated with this lung cancer screening CT technique.^4^ Thus, patient radiation exposure could be reduced by expanding the scope of LDCT lung cancer screening to assess indicators for cardiovascular disease with this single LDCT study.^5^

Calcifications in aortic and mitral valves observed on electrocardiogram (ECG)-gated calcium scoring CT (CSCT) have been shown to be manifestations of atherosclerosis and associated cardiovascular disease.^6–8^ The extent of aortic valve calcification (AVC) provides incremental value over that of coronary artery calcification (CAC) in predicting the 10-year calculated risk of coronary heart disease and is an independent predictor of cardiovascular and coronary events.^9,10^ Moreover, the presence and extent of AVC are closely related to the severity of aortic stenosis (AS),^11^ and a high aortic valve calcification score suggests the presence of severe AS that requires urgent cardiologic management.^12^ Even patients with a lesser degree of AVC should be screened for AS and monitored for disease progression.^12^ In addition, a prior study demonstrated that AVC could be measured and quantified on lung cancer screening CT with excellent reliability.^13^

In terms of indicators of cardiovascular disease, prior study suggested that the CAC score obtained from LDCT is comparable with that measured with ECG-gated CSCT.^14^ However, to the best of our knowledge, the quantified values of AVC on LDCT have not been compared with those measured on CSCT, which may serve as a reference standard for measuring AVC as in coronary artery calcium scoring.

On the other hand, the correlation of AVC extent assessed on chest CT with valvular function evaluated with echocardiography has been studied.^15,16^ Those studies concluded that there is substantial agreement between the grade of AVC on chest CT and the severity of AS at echocardiography. However, in those studies, the patients underwent contrast-enhanced chest CT or electron beam CT (EBCT) for certain indications, and the grade of AVC was assessed using a 5-point scale. No data regarding the comparison of AVC quantitative values on LDCT with AS severity assessed with echocardiography or the diagnostic performance of AVC measured on LDCT for predicting AS presence, particularly in asymptomatic subjects undergoing LDCT, have been reported. Thus, the purpose of our study was to evaluate the association between AVC on the LDCT and AS severity on echocardiography and to estimate the cutoff value of AVC on LDCT for detecting AS in asymptomatic Asian subjects.

## METHODS

### Study Population

Our institutional review board approved this retrospective study, and informed consent for using clinical data was waived. Asymptomatic subjects (without any respiratory or cardiogenic symptoms or signs) who were self-referred to the health-screening center at our institution for their general health care including lung cancer screening from 2008 through 2013 were included in this retrospective study. Both LDCT and CSCT were performed on the same day. Echocardiography was performed within 1 year of the CT scans. A total of 6338 asymptomatic subjects who had undergone LDCT, CSCT, and echocardiography were included.

### Calcium-Scoring CT

Calcium-scoring CT scan was performed using a 64-slice scanner system (Lightspeed, GE Healthcare, Waukesha, WI) and a 40-slice scanner system (Brilliance 40, Philips, Hamburg, Germany). Tube voltage was 120 kVp and tube current was 125 mA. Step and Shoot mode was used with prospectively ECG-triggered to 75% of the R-R interval in subjects with a heart rate at most 65 beats per minute (bpm) and 45% of the R-R interval in subjects with a heart rate >65 bpm. Imaging was reconstructed into a 2.5-mm slice thickness with a 512 × 512 matrix and a 25-cm field-of-view. No premedication with nitrate or beta-blocker was administered. The effective doses of the LDCT and CSCT were calculated by multiplying the given dose-length product with a conversion factor of 0.014 mSv · mGy^−1^ · cm^−1^ for adult chest CTs suggested from the American Association of Physicists in Medicine.^17^

### Low-Dose Chest CT

Chest CT was also performed using the same CT scanners (Lightspeed and Brilliance 40). Subjects remained stationary on the table between the 2 CT scans without changing position. This scan was performed with 120 kVp, 30 mAs, 0.35-second gantry rotation, and a table pitch of 1.3. The low-dose chest CT volume data were retrospectively reconstructed into a 512 × 512 matrix with a 34.5-cm field-of-view. Scans were reconstructed with an effective section thickness of 1.25 to 5.0 mm from January 2008 to September 2012 and 1.25 to 2.5 mm until the end of the study period. Measurements of AVC were obtained on 5.0-mm-thick sections from January 2008 to September 2012 and 2.5-mm-thick sections thereafter.

### Measurement of Coronary Artery and Aortic Valve Calcifications

**FIGURE 1:**
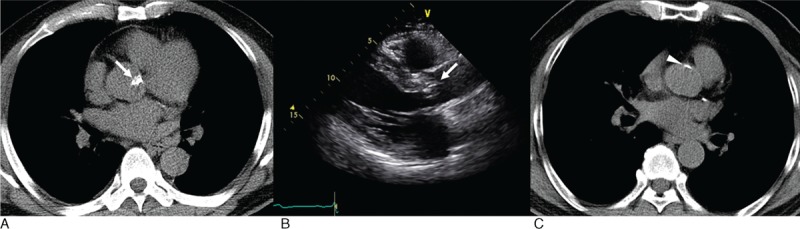
Measurement of aortic valve calcifications on representative images. (A) Low dose chest CT scan image of a 58-year-old man shows 2318.7 Agatston score of the aortic valve calcification (white arrow). (B) Corresponding echocardiography reveals calcified aortic valve leaflets (white arrow) with moderate aortic stenosis (a peak velocity of 3.2 m/sec and a mean pressure gradient of 22.1 mm Hg across the aortic valve). (C) Calcifications in the aortic sinus wall (white arrowhead) were excluded from analysis. CT = computed tomography.

Agatston calcium scores were measured using a commercial workstation (Terarecon Intuition, version, 4.4.7, TeraRecon, Inc, Foster City) with dedicated cardiac analysis software.^18,19^ High attenuation lesions, which were defined as having attenuation equal to or greater than the minimum attenuation of 130 HU, were considered to be potential calcium deposits. Calcifications were identified in the coronary arteries and aortic valve. Using the Agatston algorithm, the attenuation factor of each calcification was determined on the basis of the maximal CT attenuation of the lesion and it was as follows: factor 1 = 130 to 199 HU, factor 2 = 200 to 299 HU, factor 3 = 300 to 399 HU, and factor 4 = 400 HU or greater. The calcium score was calculated by multiplying the area of each calcified plaque by the corresponding attenuation factor.^20^ AVC was quantified using the Agatston scoring method on both LDCT and CSCT. AVC was defined as calcium within the aortic valve leaflets or aortic annulus (Figure 1A). The aortic valve was identified as the structure lying within the contiguous plane that extended from the left ventricle to the ascending aorta and was usually present in 3 or 4 consecutive images. Calcium in the aortic valve (found within the contiguous planes between the left ventricle and ascending aorta) was distinguished from coronary calcium (within the paths of the coronary arteries) by anatomic location. Calcium within the aortic sinuses, aortic wall, or both, was excluded from analysis and was not measured as AVC (Figure 1C).^21^

### Echocardiographic Evaluation

Transthoracic echocardiography measurements were performed using commercially available equipment (Acuson SC 2000; Siemens Medical Solutions USA, Inc, Mountain View, CA). Mean transvalvular gradients (mean ΔP), peak velocity (V_max_), aortic valve area (AVA) were measured. The severity of aortic stenosis was classified according to the definition of progressive AS and asymptomatic severe AS from the American College of Cardiology and American heart Association (ACC/AHA) guidelines: severe, aortic V_max_ ≥ 4 m/s or ΔP ≥ 40 mm Hg or AVA typically ≤ 1.0 cm^2^ (or AVA indexed to be body surface area [BSA] ≤ 0.6 cm^2^/m^2^); moderate, aortic V_max_ 3.0 to 3.9 m/s or mean ΔP 20 to 39 mm Hg; and mild, aortic V_max_ 2.0 to 2.9 m/s or mean ΔP < 20 mm Hg.^22^ Five sonographers, each with >5 years of experience, evaluated all cases of echocardiography.

### Assessment of Clinical Factors

At each visit, demographic characteristics, smoking status, alcohol consumption, medical history, and medication use were collected through standardized questionnaires. Smoking status was categorized into never, former, or current smokers. Height, weight, and sitting blood pressure were measured by trained nurses. Body mass index was calculated as weight in kilograms divided by height in meters squared. Hypertension was defined as a systolic blood pressure of 140 mm Hg or more, a diastolic blood pressure 90 mm Hg or more, a self-reported history of hypertension, or current use of antihypertensive medications. Hyperlipidemia was defined as total cholesterol at least 240 mg/dL or use of cholesterol-lowering medication. Diabetes mellitus was defined as a fasting serum glucose at least 126 mg/dL, a self-reported history of diabetes, or current use of antidiabetic medications. Serum total cholesterol, high-density lipoprotein cholesterol, low-density lipoprotein cholesterol, serum levels of fasting blood sugar, HbA1c, were measured in fasting blood samples collected after at least 12 hours of fasting.

### Statistical Analysis

Descriptive statistics are provided as mean and standard deviation values. The relationship between AVC scores measured on LDCT and on CSCT was analyzed using the paired *t* test and bivariate Spearman rank correlation test. The correlations between degree of AS on echocardiography and the quantified AVC on both LDCT and CSCT were analyzed using the bivariate Spearman rank correlation test. The diagnostic performance of the AVC scores on LDCT and CSCT for detecting AS in subjects with AVC was evaluated by receiver operating characteristic curve analyses and by adopting echocardiography as the reference standard. Univariate and multivariate logistic regression was used to evaluate clinical factors associated with AS and AVC.

Because 2 different scanners and 2 kinds of slice thickness were used for acquiring CT images, the effects of scanner and slice thickness in the detection of AS were analyzed using univariate analysis. In addition, the effect of different slice thickness on the correlation between the extent of AVC score and AS parameters was analyzed using Fisher z transformation.

We used SAS version 9.4 and R version 3.1.2 for statistical analysis. All statistical tests were two-sided, and significance was set at *P* < 0.05.

## RESULTS

### Baseline Characteristics

The subject characteristics according to the presence or absence of AVC are shown in Table 1. The interval between LDCT and echocardiography was 1.4 ± 3.7 months (range, 0–12 months). Persons with AVC were significantly more likely to be older and have a high prevalence of hypertension, diabetes, and dyslipidemia. In addition, they tended to have higher CAC scores in comparison with those without AVC (Table 1). In contrast, there were no significant differences in age, sex, or cardiovascular risk factors between 2 groups, when subjects with AVC were divided according to the presence or absence of AS (Table 2). The mean effective radiation doses on LDCT and CSCT were 0.7 ± 0.3 and 0.6 ± 0.8 mSv, respectively.

**TABLE 1:**
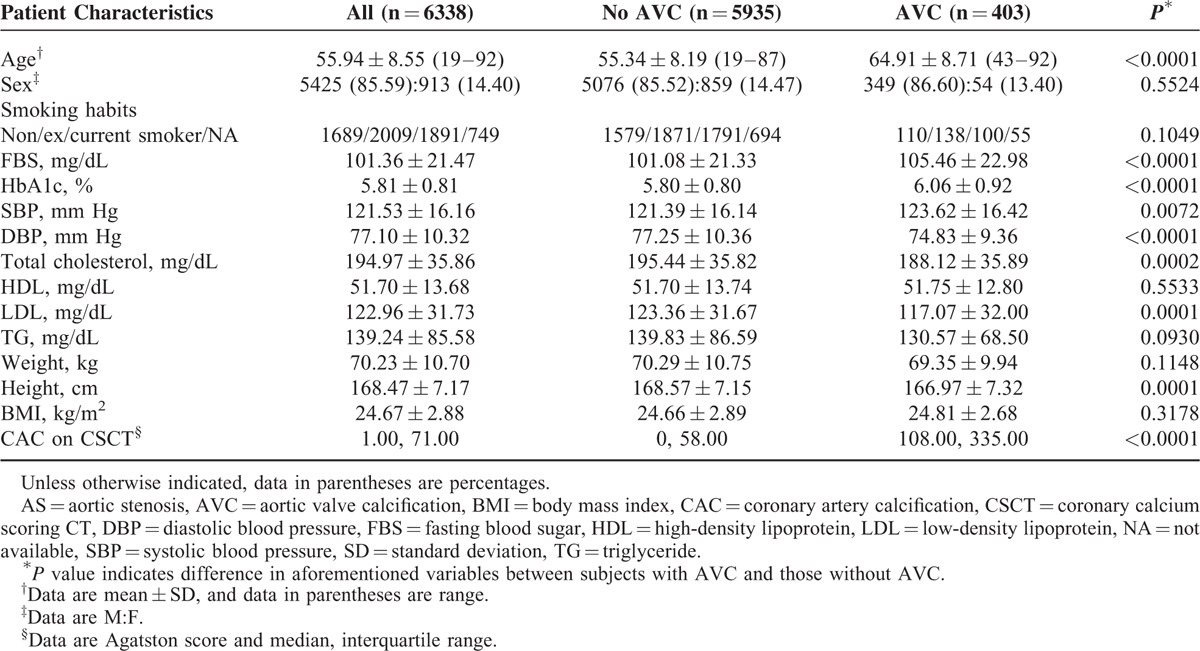
Baseline Characteristics of All Subjects and Subgroups by Presence and Absence of AVCs

**TABLE 2:**
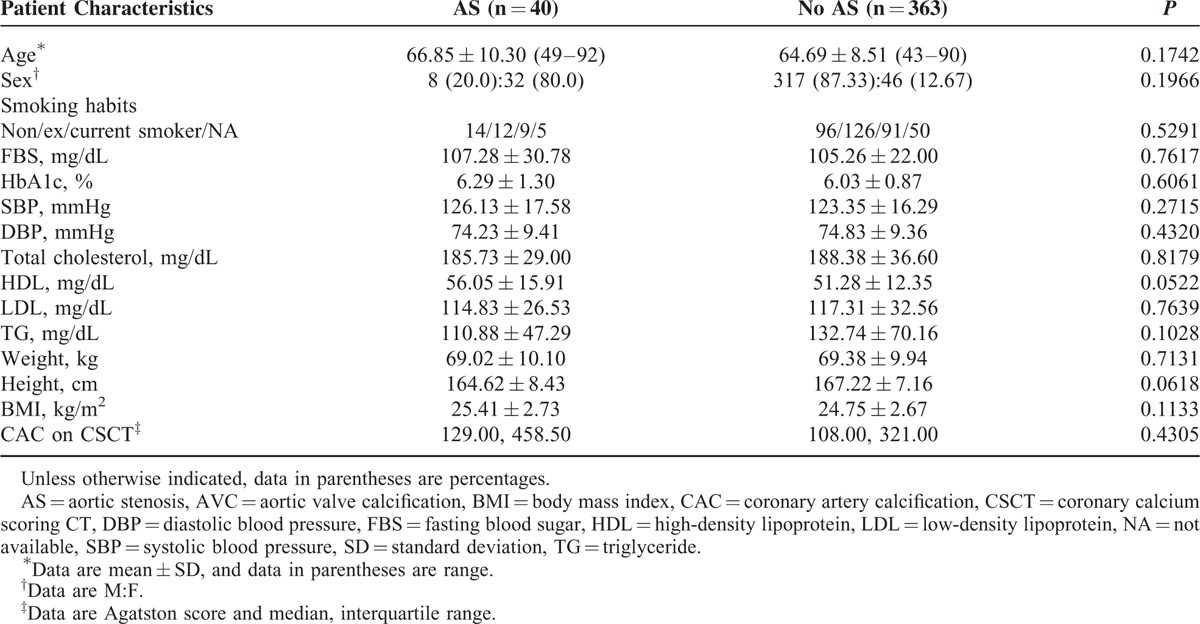
Baseline Characteristics Grouped by Presence and Absence of Aortic Stenosis in Subjects With AVCs

### Clinical Factors Associated With AVC and AS

Significant clinical factors associated with AVC in the univariate analysis were age, extent of CAC on CSCT, diabetes mellitus, hyperlipidemia, and hypertension. Age (odds ratio [OR] = 1.10, 95% CI: 1.09–1.12), extent of CAC on CSCT (OR = 1.38, 95% CI: 1.28–1.48), and hypertension (OR = 1.39, 95% CI: 1.10–1.76) retained statistical significance on a multivariate analysis.

Identified significant clinical factors associated with AS in subjects with AVC by univariate analysis were extent of AVC on LDCT and CSCT. By contrast, the extent of CAC on CSCT was not associated with AS in subjects with AVC on a univariate analysis. Extent of AVC on LDCT was the only significant clinical factor associated with AS on subsequent multivariate analyses (OR = 106.66, 95% CI: 16.56–687.04).

### Relationship Between LDCT and CSCT and Severity of AS on Echocardiography

In the evaluation of AVC on LDCT, 403 (6.4%) of 6338 subjects were noted to have AVC. The median AVC score on LDCT was 58.91 (interquartile range, 21.42–133.97).

**FIGURE 2:**
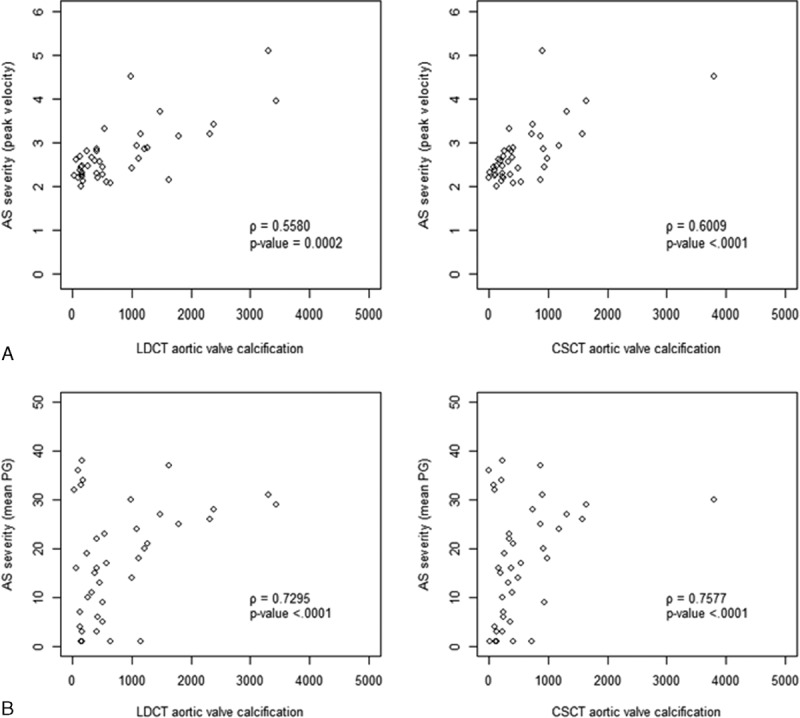
Correlation analyses of aortic valve calcification scores and echocardiography parameters of peak velocity and mean pressure gradient (aortic valve calcification scores from low-dose CT and calcium-scoring CT). (A) Correlation analysis for peak velocity (m/s) (Spearman rank correlation coefficient: *r* = 0.58, *P* < 0.0001; *r* = 0.60, *P* < 0.0001 with aortic valve calcium scores at low-dose CT and calcium-scoring CT, respectively). (B) Correlation analysis for mean pressure gradient (mm Hg) (Spearman rank correlation coefficient: all *r* = 0.76; all *P* < 0.0001 with aortic valve calcium scores at low-dose CT and calcium-scoring CT). CT = computed tomography.

**FIGURE 3:**
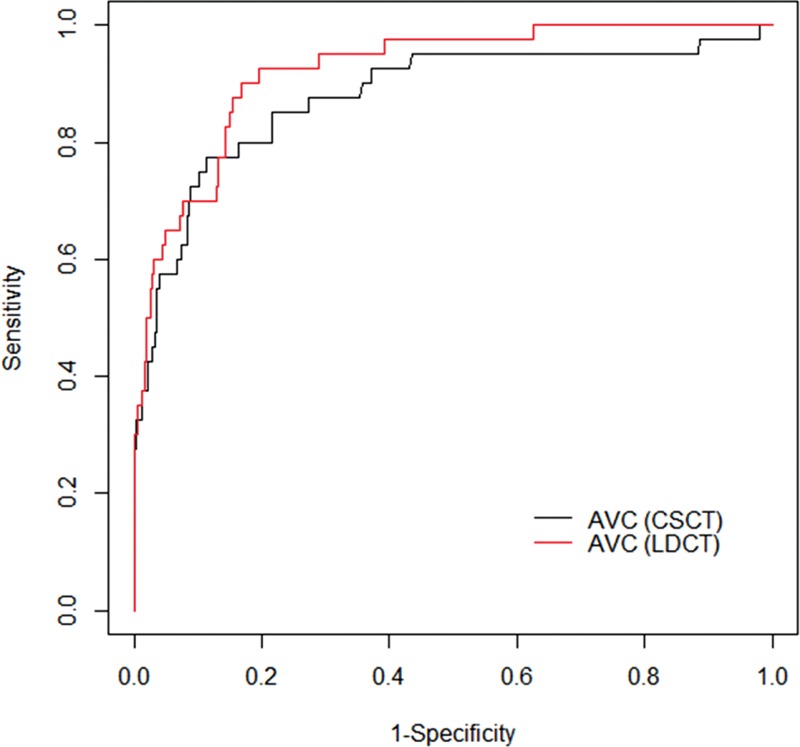
Cutoff values and diagnostic performance of quantitative AVC to predict AS. ROC curve for Agatston AVC scores to predict AS on LDCT (red line) and CSCT (black line). This analysis indicates that an Agatston AVC score >138.37 on LDCT had the optimal combination of sensitivity (90.0%) and specificity (83.20%) for identifying subjects with aortic stenosis. AS = aortic stenosis, AVC = aortic valve calcification, CSCT = coronary calcium scoring CT, LDCT = lung cancer-screening low-dose CT.

Among subjects with AVC, 40 (10%) were identified to have AS on echocardiography and the degree of the AS was as follows: mild-degree AS, 31; moderate-degree AS, 6; and severe-degree AS, 3. Of the 40 subjects, 3 had bicuspid valve: 2 with mild AS and 1 with severe AS. The AVC score on LDCT showed a positive correlation with peak velocity (Figure 2A) and mean pressure gradient (Figure 2B) (*r* = 0.58, *P* < 0.001; *r* = 0.76, *P* < 0.001 for peak velocity and pressure gradient, respectively) of the aortic valve. Median and interquartile range of AVC on LDCT were as follows: subjects with AVC but no AS ([n = 363], 48.2, 19.11–107.14); those with mild AS ([n = 31], 369.73, 162.43–567.54); those with moderate AS ([n = 6], 1636, 1001.32–2335.85); those with severe AS ([n = 3], 3302.7, 2046.3–3433.4). On the review of echocardiography, only 4 subjects without AVC had mild AS and 1 of them had bicuspid valve. The extent of AVC was significantly larger (*P* < 0.001) in 40 subjects with AS (median, 447.67; interquartile range, 171.70–1184.75) compared with those without AS (median, 48.20; interquartile range, 19.11–107.14). The diagnostic estimates for detecting AS using AVC score on LDCT were as follows: sensitivity, 90.0%; specificity, 83.2%; positive predictive value (PPV), 37.1%; negative predictive value (NPV), 98.6%; area under the curve (AUC), 0.92; with optimal cutoff value of AVC, 138.37 (Figure 3).

The median AVC score on CSCT was 76.29 (interquartile range, 40.51–140.87). The AVC score on CSCT correlated positively with peak velocity and mean pressure gradient (Figure 2) (*r* = 0.60, *r* = 0.76; all *P* < 0.001). The extent of AVC was significantly heavier (*P* < 0.001) in subjects with AS (median, 348.19; interquartile range, 207.13–872.81) compared with those without AS (median, 66.96; interquartile range, 35.96–120.34). The diagnostic estimates for detecting AS using AVC score on CSCT were as follows: sensitivity, 77.5%; specificity, 88.7%; PPV,43.0%; NPV, 97.2%; AUC, 0.88; with optimal cutoff value of AVC, 198.88 (Figure 3). AVC score measured from LDCT showed a strong positive correlation with that from CSCT (*r* = 0.83, *P* < 0.001).

### Different CT Scanner and Slice Thickness in AVC Quantification

The quantified values of AVC on LDCT according to scanner type and slice thickness are shown in Table E1. In the detection of AS using AVC score, scanner type and slice thickness were not significant factors on a univariate analysis (*P* = 0.19 and *P* = 0.97, respectively). In the correlation of AVC score and AS parameters, correlation coefficients were acquired from 37 patients who adopted the same CT scanner (scanner type II, Table E2). When 2 (2.5 and 5.0 mm) kinds of slice thickness were used in CT scanning, the correlation between the extent of AVC score and AS parameters was not different between different slice-thickness groups (peak velocity and mean pressure gradient; *P* = 0.73 and *P* = 0.85, respectively; using Fisher z transformation).

## DISCUSSION

In the current study, the quantification of AVC from LDCT is well correlated with the value obtained using dedicated CSCT. We also found that quantified values of AVC from LDCT show good correlation with the hemodynamic degree of AS evaluated with echocardiography. Although age, extent of CAC on CSCT, and hypertension retained statistical significance in association with AVC, the extent of AVC on LDCT was the only significant clinical factor associated with AS. Therefore, LDCT for lung cancer screening can be used to detect the presence of AVC and to quantify the amount of AVC for diagnosing AS in asymptomatic subjects undergoing LDCT for lung cancer screening. Our results are based on a screening population of substantial sample size. Moreover, quantitative measures using the Agatston method for AVC are adopted in all subjects.

The current study also provides data describing the relationship of AVC scores between LDCT and CSCT. Previous studies have focused on the comparison of coronary calcium scoring between ungated LDCT and ECG-gated CSCT.^14,23^ The study showed good correlation (*r* = 0.89–0.96) in coronary calcium scoring between the 2 CT scan protocols. Our data showed that the AVC score of ungated LDCT correlates well with that of ECG-gated CSCT. Although we did not analyze the reproducibility of the quantification, the reproducibility of AVC measurement was reported to allow serial investigations over a time suitable for clinical follow-up.^24^

It is also known that there is a close correlation (*r* = 0.54) between echocardiographic measures of aortic stenosis and AVC scores measured by nongated helical CT in patients with aortic stenosis. Using different CT techniques (multislice or EBCT) and different assessment (visual or Agatston units), AVC at nongated imaging has been shown to have a definite and nonlinear correlation with echocardiographic measures of AS.^16,25–27^ Our study corroborates the results of previous studies by demonstrating that the severity of AS evaluated with echocardiography correlates well with that of LDCT Agatston-based scoring of AVC.

Koos et al^16^ reported that AVC is an incidental finding in 18% of patients undergoing multidetector row CT performed for various clinical indications other than aortic valve disease. Hunold et al^28^ suggested that AVC is incidentally found in 23% of 1812 consecutive patients with known or suspected coronary artery disease. In our study, the incidence of AVC on LDCT was relatively low compared with those in previous studies.^16,28^ The reasons for this lower incidence are presumably the following: our cohort was composed of asymptomatic subjects undergoing LDCT for lung cancer screening and 2 previous studies regarding the prevalence of AVC were performed in the United States and Germany, respectively, where the prevalence of AVC may be different from that (403 of 6338 subjects, 6.4%) in an Asian country.

Shavelle et al^21^ concluded that an AVC Agatston score >150 at ECG-gated EBCT may warrant echocardiographic evaluation, and an AVC Agatston score >500 at EBCT should lead to echocardiographic assessment for AS. Cowell et al^12^ suggested that a threshold AVC Agatston score >3700 is sufficient to make a diagnosis of severe AS on unenhanced CT scans. Our cutoff value of 138.37, which was for screening AS in an asymptomatic group, Agatston score is lower than those of previous studies. In our AS group, mild AS was present in 77.3% of patients, and severe AS in 6.8%. To the best of our knowledge, only few studies have been performed regarding the cutoff values of AVC for diagnosing AS in an asymptomatic group. Moreover, although AVC score measured from LDCT showed a strong positive correlation with that from CSCT, ungated LDCT generally yielded lower scores than ECG-gated CSCT, especially in subjects without AS and mild AS. This lowering tendency in LDCT may have contributed to the lowered cutoff values of AVC for the prediction of AS compared with those in CSCT. The reason for this difference in AVC scores between LDCT and CSCT might be caused by different slice thickness and motion artifact in LDCT scanning. In our study, the presence of AS was shown to be related more to the extent of AVC than conventional coronary risk factors. Previous studies have also suggested that none of the “traditional” major coronary risk factors show significant correlation with AS.^29,30^

Our study has several limitations. First, all subjects were self-referred to health-promotion center, suggesting that our study may have a selection bias as a large confounding factor. Second, because of the 1-year interval between echocardiography and CT examination, the AS may have progressed. However, we judged this to be a minor problem as most subjects had mild AS (79.5%) and progression of AS is typically slow. The reported rate of reduction in aortic valve area is 0.10 ± 0.27 cm^2^ or 7 ± 18% per year.^31^ Nevertheless, echocardiography obtained at the similar time to CT would have contributed to evaluating the exact relationship of AS on echocardiography and AVC extent on CT. Third, even though we included subjects with all grades of AS (ranging from mild stenosis of the valve area to severe hemodynamic impairment), the majority of the subjects had a mild degree of AS (79.5%). Therefore, it was impossible to calculate the optimal cutoff values for the prediction of moderate and severe AS that needs intervention or valve replacement surgery. Fourth, we used 2 different CT scanners, which may have resulted in measurement discrepancies. However, previous studies have concluded that overall reproducibility and agreement between cardiac CT scans is sufficiently high to allow for serial assessment of AVC.^13,24^ In the current study, 57 (14.1%) of 403 patients with AVC and 3 (7.5%) of 40 subjects with AS diagnosed on echocardiography underwent CT study with a different CT scanner. These patients constituted a relatively small group compared with a remaining large group of subjects. Our data showed different type of CT scanner did not affect the detection rates of AS. The final limitation may be the adoption of 2 different (2.5 and 5.0-mm) slice thicknesses for image reformation in LDCT scanning. With thick-section (5.0-mm-section thickness) images, the AVC might have been measured smaller than that with thinner-section (2.5-mm-section thickness) images mainly owing to partial-volume averaging effect especially in subjects with mild burden of AVC. However, our data demonstrated that slice thickness did not affect the detection of AS or the correlation between AVC score on LDCT and AS parameters on echocardiography.

## CONCLUSIONS

Because AVC extent on LDCT is the significant clinical factor related to the presence of AS, echocardiography is recommended for screening AS based on quantified AVC values with the threshold of 138.37 on LDCT in asymptomatic Asian subjects. LDCT for lung cancer screening, performed in high-risk smokers (>30 pack years and <15 years after smoking cessation) who also have the potential for coronary artery and aortic valvular heart disease, should be read for the presence and extent of AVCs, because the LDCT provide image data for detecting and quantifying AVC.

## Supplementary Material

Supplemental Digital Content
