# Supplementary material for: Quantification of Aortic Valve Calcifications Detected During Lung Cancer-Screening CT Helps Stratify Subjects Necessitating Echocardiography for Aortic Stenosis Diagnosis
Source: Medicine (Baltimore). 2016 May 13;95(19):e3710. doi: 10.1097/MD.0000000000003710 (PMC4902555; doi:10.1097/MD.0000000000003710)
Supplement: Supplemental Digital Content [file medi-95-e3710-s001.doc]

**Table E1** Quantification of Aortic Valve Calcification on LDCT according to Scanner Type and Slice Thickness

|  | Scanner | Slice thickness | N | Median | Interquartile range |
| --- | --- | --- | --- | --- | --- |
|  | I | 2.5 mm | 23 | 27.99 | 11.08 –56.10 |
| Total | I | 5 mm | 36 | 31.58 | 16.45 – 96.25 |
| (N = 403) | II | 2.5 mm | 109 | 63.85 | 21.42 – 152.21 |
|  | II | 5 mm | 235 | 66.54 | 24.72 – 142.10 |
|  | I | 2.5 mm | 1 | 329.46 | NA |
| AS | I | 5 mm | 2 | 591.46 | 29.41 – 1153.5 |
| (N = 40) | II | 2.5 mm | 12 | 437.35 | 222.37 – 823.67 |
|  | II | 5 mm | 25 | 512.71 | 171.66 – 1625.70 |

Abbreviations: AS = aortic stenosis, LDCT = lung cancer-screening low-dose CT, NA = not applicable.

**Table E2** Correlation between AVC Score on LDCT and AS Parameters on Echocardiography in AS Patients

|  | Scanner | Slice thickness | N | P†  (vs. peak velocity) | P†  (vs. mean PG) |
| --- | --- | --- | --- | --- | --- |
|  | I | 2.5 mm | 1 | NA | NA |
| AS | I | 5 mm | 2 | 1.000 | NA |
| (N = 40) | II | 2.5 mm | 12 | 0.518 | 0.769 |
|  | II | 5 mm | 25 | 0.610 | 0.737 |

† P values were calculated using spearman correlation analysis.

Note – Peak velocity and mean PG were acquired from echocardiography.

Abbreviations: AS = aortic stenosis, AVC = aortic valve calcification, LDCT = lung cancer-screening low-dose CT, NA = not applicable, PG = pressure gradient.
